# Impact of stimulus uncanniness on speeded response

**DOI:** 10.3389/fpsyg.2015.00662

**Published:** 2015-05-21

**Authors:** Kohske Takahashi, Haruaki Fukuda, Kazuyuki Samejima, Katsumi Watanabe, Kazuhiro Ueda

**Affiliations:** ^1^Research Center for Advanced Science and Technology, The University of TokyoTokyo, Japan; ^2^Core Research for Evolutional Science and Technology, Japan Science and Technology AgencyTokyo, Japan; ^3^Graduate School of Arts and Sciences, The University of TokyoTokyo, Japan; ^4^Brain Science Institute, Tamagawa UniversityTokyo, Japan; ^5^Faculty of Science and Engineering, Waseda UniversityTokyo, Japan

**Keywords:** Uncanny, speeded response, reaction Time, direction discrimination, spatial cueing, dot-probe, uncanny valley

## Abstract

In the uncanny valley phenomenon, the causes of the feeling of uncanniness as well as the impact of the uncanniness on behavioral performances still remain open. The present study investigated the behavioral effects of stimulus uncanniness, particularly with respect to speeded response. Pictures of fish were used as visual stimuli. Participants engaged in direction discrimination, spatial cueing, and dot-probe tasks. The results showed that pictures rated as strongly uncanny delayed speeded response in the discrimination of the direction of the fish. In the cueing experiment, where a fish served as a task-irrelevant and unpredictable cue for a peripheral target, we again observed that the detection of a target was slowed when the cue was an uncanny fish. Conversely, the dot-probe task suggested that uncanny fish, unlike threatening stimulus, did not capture visual spatial attention. These results suggested that stimulus uncanniness resulted in the delayed response, and importantly this modulation was not mediated by the feelings of threat.

## Introduction

Uncanniness, or the feeling that something is familiar yet at the same time strange, is elusive. While we certainly experience feelings of uncanniness in daily situations, the causes of those feelings and their effects on our behavior remain unclear. Feelings of uncanniness have recently attracted increasing attention, particularly in the field of human–robot interaction. For example, the “uncanny valley” (Mori, 1970) is now a widely known concept. While people do not perceive uncanniness when robots look either entirely human or not like humans at all, they experience feelings of uncanniness, or at least negative emotion, when faced with humanoid robots whose appearances are similar to those of humans but whose motions are dissimilar to those of humans. Several theoretical explanations have been proposed regarding the causes of the uncanny valley (e.g., prediction error, Saygin et al., 2012; fear of mortality, MacDorman and Ishiguro, 2006). However, the nature of uncanniness and uncanny valley is far from conclusive. The explanations above have not been empirically verified; there are also conflicting evidences regarding the uncanny valley phenomenon (Looser and Wheatley, 2010; Yamada et al., 2012; Burleigh et al., 2013; Cheetham et al., 2014).

Compared to the causes of uncanniness, the behavioral effect of uncanniness, or what occurs at a behavioral level when people experience feelings of uncanniness, has been understated. However, emotion regulates perception and behavior (Zadra and Clore, 2011). For example, positive emotions broaden visual spatial attention (Fredrickson and Branigan, 2005). Nittono et al. (2012) showed that a feeling of *kawaii* (a Japanese word meaning “cute”) narrows attention and leads to careful behavior. In addition, threatening objects capture visual spatial attention and enhance behavioral performance (Fox et al., 2000; Ohman et al., 2001). Revealing the behavioral effects of uncanniness is also important in terms of the uncanny valley phenomenon, since it can provide an objective measure of the observers’ reaction facing the uncanny valley. Therefore, in keeping with these studies, the purpose of the present study is to investigate the correlation between behavioral performance and feelings of uncanniness via psychophysical experiments.

The present study focused on the effect of uncanniness of visual stimuli on reaction times (RTs) in various speeded response tasks, with two possible outcomes. Visual stimuli that evoke strong feelings of uncanniness could speed up perceptual processes. For example, threatening objects capture visual spatial attention more efficiently than do non-threatening objects, which leads to faster detection of targets in visual search or dot-probe paradigms (Fox et al., 2000; Ohman et al., 2001, but see also Quinlan, 2013). This has obvious ecological and evolutional consequences, given the need to detect and avoid threats as quickly as possible to maximize our chances of survival. As feelings of uncanniness are suggested to be associated with fear (MacDorman and Ishiguro, 2006), uncanny stimuli might be detected quickly and processed more rapidly relative to other stimuli. However, visual stimuli associated with feelings of uncanniness may delay speeded responses. For example, it was suggested that prediction error underlies the uncanny valley phenomenon (Saygin et al., 2012). In accordance with this hypothesis, In a Posner-like cueing paradigm, RTs for target detection were longer with uncanny facial cues (Chaminade and Okka, 2013). More generally, negative stimuli sometimes lead to increased RTs (Derryberry, 1991; Leppänen et al., 2003), which are associated with modulation of attentional and arousal factors (Yechiam and Hochman, 2013). Given these two possible outcomes, revealing the correlation between RTs and feelings of uncanniness would be the first step toward understanding the nature of those feelings.

The previous studies have examined RT in terms of the uncanny valley phenomenon. For example, ambiguous stimuli took longer RT in categorization (Cheetham et al., 2014), and the RT in categorization negatively correlated with the stimulus likability (Yamada et al., 2012). However, these studies could not address the relation between the uncanniness and RT, since both were related to categorical ambiguity. To investigate the behavioral effects of uncanniness, in the present study, we used pictures of fish as experimental stimuli. Fish pictures were suitable for our purposes for four reasons. First, fish has no categorical ambiguity. Second, the appearance of fish evokes a wide range of feelings of uncanniness, and this facilitates the quantitative examination of the effects of uncanniness. Third, the figural properties of fish are uniform, and fish have obvious directional information, which are ideal properties for stimuli in speeded response tasks. Forth, use of a fish stimulus allows the avoidance of some experimental confounding. For example, if we use real human faces vs. android faces as experimental stimuli, stimulus category or animacy perception could covary with uncanniness. These confounding lead to difficulty in revealing the nature of feelings of uncanniness. Thus, fish seems to be suitable to investigate the behavioral effects of uncanny feeling *per se*, avoiding potential experimental confounding by using human face or human-like agents as a stimulus. However, we should be also aware of the limitation of the present study, that is, how the uncanniness of fish directly related to the uncanniness in the uncanny valley phenomenon. Someone might consider the uncanniness of fish and uncanniness of human-like robot might differ qualitatively (e.g., visually evoked uncanniness vs. socially motivated uncanniness). We mentioned this issue again in Section “General Discussion.”

In a pilot experiment, we asked participants to rate the eeriness of several pictures of fish and determined the stimulus set for the psychophysical experiments. Experiment 1 investigated the effects of uncanniness on manual responses directed toward the stimuli. Participants indicated the direction toward which fish were oriented (left or right) as quickly as possible. In Experiment 2, we adapted a directional-cueing paradigm (endogenous attention task, Posner et al., 1980). Participants were asked to indicate the location of a peripheral target (left or right hemifield) as quickly as possible. A task-irrelevant fish picture was presented shortly before target onset. This task allowed us to examine the indirect effect of uncanny stimuli on manual responses to a subsequent target. In Experiment 3, we used a dot-probe task to investigate whether uncanny fish pictures would capture visual spatial attention and facilitate the detection of a subsequent target at the preceding stimulus location.

## Materials and Methods

### Participants

Graduate and undergraduate students of universities around Tokyo area participated in the pilot experiment as a paid volunteer (1,000 JPY per hour). They have reported normal or corrected-normal visual acuity and no neurological or psychiatric illness. They had no specific knowledge about the uncanny valley phenomenon. All participants in the present study provided written informed consent. The study was approved by the Ethics Committee of the University of Tokyo and run in accordance with the Declaration of Helsinki.

### Stimuli and Apparatus

**FIGURE 1:**
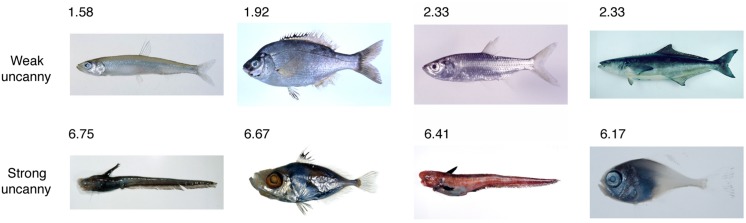
**Sample fish pictures.** The top and bottom rows show the weak and strong uncanny pictures of fish, respectively. The numerical values indicate mean uncanniness ratings on a seven-point scale. ©Kanagawa Prefectural Museum of Natural History (Photo: Hiroshi Senou).

We collected 180 colored pictures containing whole bodies of left-oriented fish from the FishPix academic database (**Figure 1**. Kanagawa Prefectural Museum of Natural History, Odawara, and the National Museum of Nature and Science, http://fishpix.kahaku.go.jp/fishimage-e/index.html). The database have more than 80,000 fish pictures, and we chose the fish stimuli based on the following criteria: (1) pictures depict whole body, (2) a creature depicted in the picture obviously looks a fish, and (3) direction of a fish is clear. As well, we chose the stimuli so that the uncanniness of the fish varies as largely as possible. One of the assistants who did not know the purpose of the study performed this selection.

The experiments were conducted in a quiet and dimmed lit room. Stimulus presentation and response acquisition was controlled by Mac-mini with Matlab and Psychtoolbox 3 toolbox (Brainard, 1997; Pelli, 1997) and a 21-inch CRT monitor (100 Hz, viewing distance was approximately 57 cm).

## Pilot Experiment

To confirm that the uncanniness of fish pictures varied sufficiently and obtain an uncanniness rating for each fish picture, we conducted a pilot experiment in which participants rated the eeriness of fish. In addition, we asked the participants to rate the familiarity and deliciousness of fish. In the uncanny valley phenomenon, uncanniness is often associated with unfamiliarity and negative emotion (MacDorman and Ishiguro, 2006; Yamada et al., 2012). Therefore, we determined whether the uncanniness of fish was negatively correlated with familiarity and deliciousness.

### Methods

Twelve participants (seven male and five female, mean age was 22.3) were asked to rate the eeriness (uncanniness)^1^, familiarity, and deliciousness (in terms of taste) of the fish on a seven-point scale (1 = “not at all” and 7 = “very strongly”). For each trial, a picture of a fish (640 × 640 pixel, 23° × 23°) was presented at the center of the screen. Scoring scales for three questions were presented below the stimulus picture. The participants viewed the pictures of fish freely and indicated their ratings using a mouse. After rating the fish according to the three questions, the subsequent fish picture appeared. We presented each participant with 180 pictures.

### Results

The average eeriness rating for fish pictures ranged widely from 1.58 to 6.75 (**Figure 1**). Therefore, the fish pictures were suitable for quantitative examination of the behavioral effects of uncanniness. While the uncanniness of some fish was consistent between participants, some pictures showed large individual differences. In order to reduce the individual differences in the psychophysical tasks, we excluded pictures with large SD (SD > 1.53) and used 103 of the original 180 pictures in subsequent experiments. The 103 pictures were divided according to strength of uncanniness rating as follows: weak (32 pictures, mean = 2.43), medium (31 pictures, mean = 4.13), and strong (40 pictures, mean = 5.68). **Figure 1** contains examples of fish with weak and strong uncanniness ratings.

**FIGURE 2:**
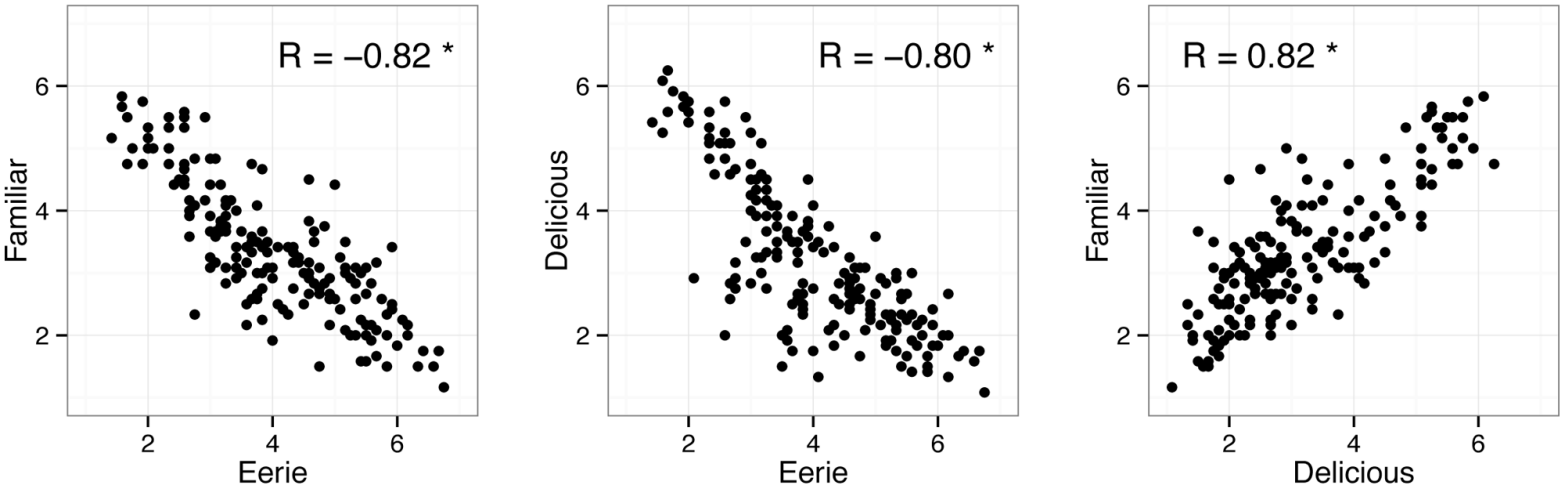
**Scatterplot and correlations between eeriness, familiarity, and deliciousness ratings.** Data points indicate individual fish pictures. The ‘∗’ indicates that the correlation coefficient was statistically significant.

**Figure 2** shows the correlations between the three question items. As expected, eeriness (mean = 4.06, SD = 1.91) was negatively correlated with both familiarity (mean = 3.34, SD = 1.98) and deliciousness (mean = 3.18, SD = 1.79). Therefore, fish rated as eerie were also rated unfamiliar and evoked negative emotion, which was consistent with previous studies examining the uncanny valley phenomenon (MacDorman and Ishiguro, 2006; Yamada et al., 2012).

## Experiment 1

In Experiment 1, we investigated the effect of the uncanniness of fish pictures on RTs on a direction discrimination task, in which participants indicated the heading direction of a fish as quickly as possible.

### Methods

**FIGURE 3:**
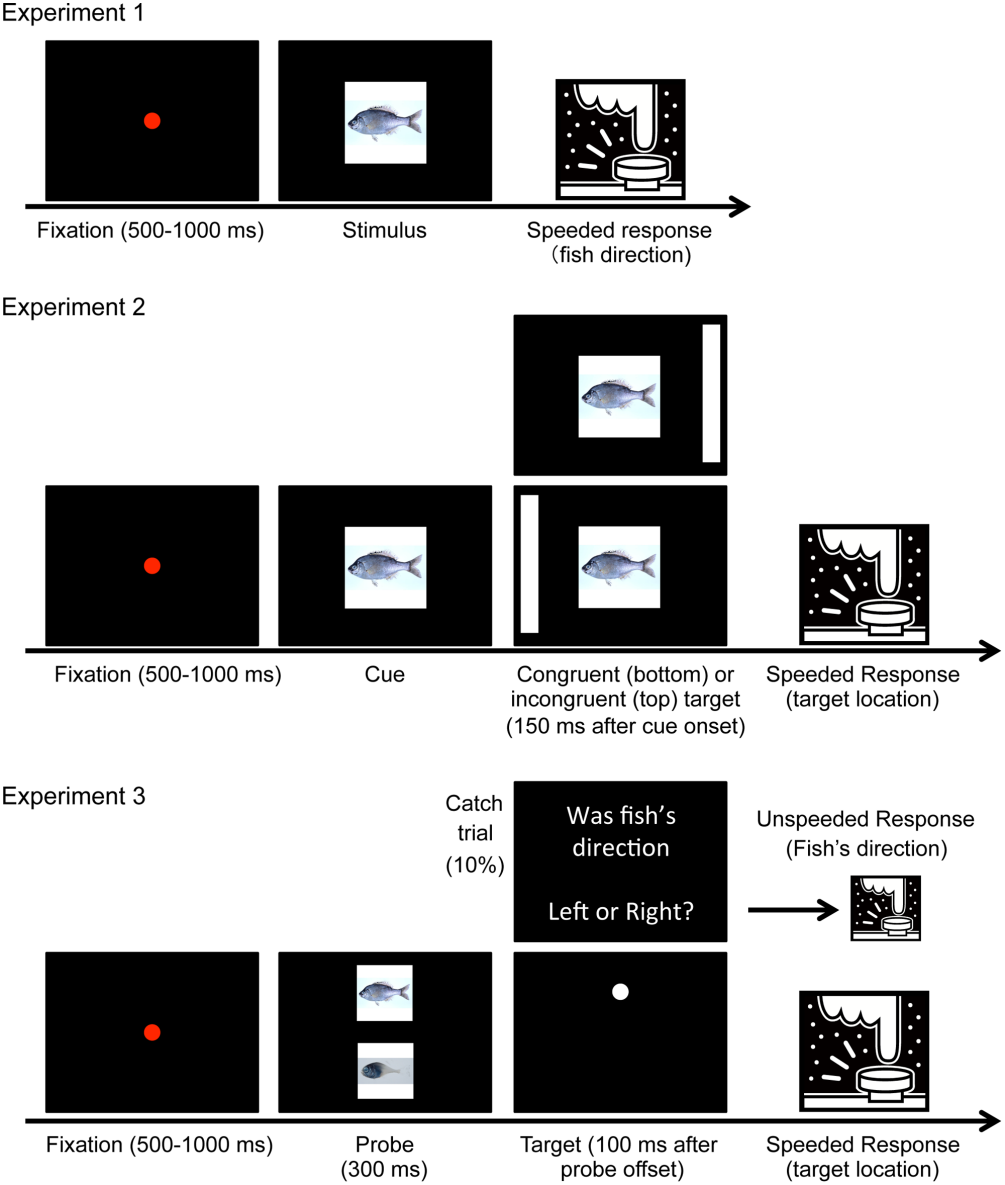
**Trial sequences of experiments.** In Experiment 2, cue congruency was randomly determined for each trial. In Experiment 3, 12 catch trials and 120 target detection trials were presented in a random sequence.

Twenty-five volunteers (17 male and 8 female, mean age was 21.2) participated in Experiment 1. **Figure 3** depicts the trial sequences. Visual stimuli were presented on a 21-inch CRT monitor (100 Hz). The pictures (23° × 23°) were presented at the center of the screen. Each trial began with the presentation of a red fixation point (duration: 500–1000 ms, randomly determined) followed by a fish picture, which was presented in a random orientation (left or right) until a response was provided. The participants indicated the direction toward which the fish was oriented by pressing the “F” or “J” key as quickly and accurately as possible. RTs were defined as the time that elapsed from target onset to key press. Each fish picture was presented once (totaling 103 trials).

### Results and Discussion

**FIGURE 4:**
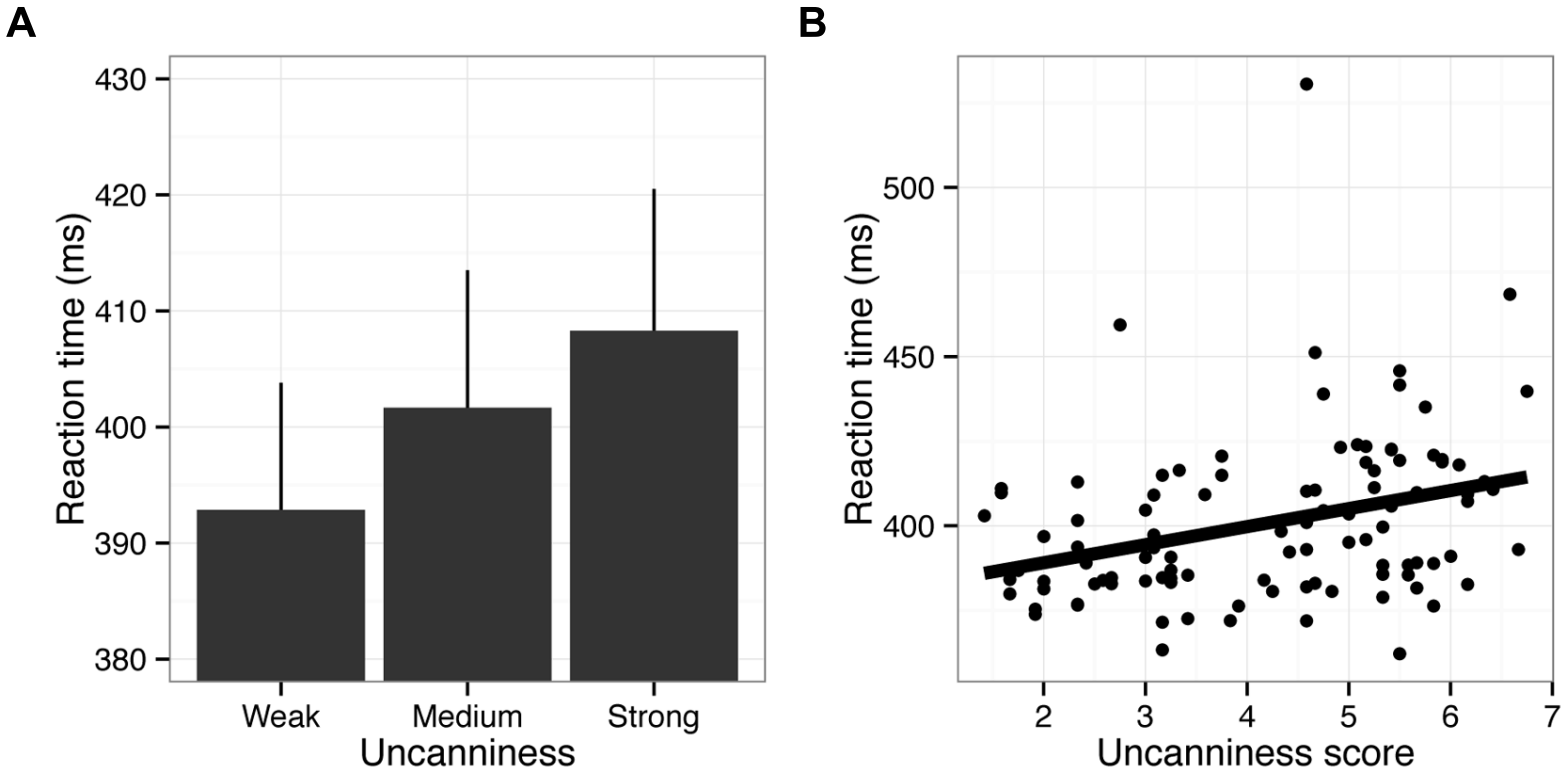
**Results of Experiment 1. (A)** RTs are shown as a function of the strength of uncanniness. Error bars indicate SE of mean (*N* = 25). **(B)** Scatter plot of RTs and uncanniness scores. Each data point indicates an individual fish picture averaged across participants. The linear regression line is also displayed. Note that there may have been an outlier, as shown in **(B)**. The scientific name for this fish is *Boulengerella maculate*, of which body shape may be atypical. However, removing data regarding this fish from the analysis did not affect the results.

Trials with incorrect responses (1.17%) and outliers (RTs shorter or longer than mean ± 3SD, 0.97%) were excluded from the RT analysis. We found that pictures with stronger uncanniness led to longer RTs (**Figure 4A**), *F*(2,48) = 9.37, *p* < 0.001, $\eta_{p}^{2}$ = 0.28. Paired comparisons showed that RTs in the weak uncanny condition were significantly shorter than were those in other conditions (correction for multiple comparisons using Ryan’s method: *p* < 0.05). In addition, the uncanniness scores in the pilot experiment and RTs in Experiment 1 were positively correlated (**Figure 4B**; *r* = 0.39, *p* < 0.01). Note that speed-accuracy tradeoff was of no consequence here, as strength of uncanniness did not influence error rates, *F*(2,48) = 0.39, *p* = 0.67, $\eta_{p}^{2}$ = 0.02. Therefore, the results clearly demonstrated that strong uncanniness in a stimulus delays directional discrimination of that stimulus. One possible confounding might be due to our stimulus selection; for example, the uncanny fish might have ambiguity in direction. However, this is unlikely happened since we used only the fish pictures whose direction is clear and as a result we did not observe the effects on the error rate.

## Experiment 2

Results from Experiment 1 suggested that the uncanny fish pictures delayed responses in the direction discrimination task. Direction discrimination required explicit processing of the fish stimuli, and it was unclear whether responses were only delayed when this requirement was present. Experiment 2 attempted to extend the results of Experiment 1 to indirect processes. For this purpose, we adapted a direction cueing paradigm, in which participants indicated the target location as quickly as possible while a directional cue was presented immediately before the target, and used the fish pictures as task-irrelevant directional cues. If the uncanniness of the fish pictures only delayed processes that were explicitly related to the stimuli, it should have had no effect on the cueing task. However, feelings of uncanniness might automatically have affected concurrent or subsequent perceptual processes, even when processing uncanny stimuli was not required for the task. In this case, we would expect to find a delay in target detection, due to the presentation of uncanny cues.

### Methods

The same twenty-five volunteers as Experiment 1 participated in this experiment. The cue stimulus was a picture of left- or right-oriented fish or a gray square (23° × 23°). The target stimulus was a white rectangle (1.5° × 36°) presented at the left or right side of the screen (24° away from the fixation).

**Figure 3** shows the trial sequence. Each trial began with the presentation of a red fixation point for 500–1000 ms followed by the randomly oriented (left or right) cue stimulus. The target stimulus was presented in the left or right position 150 ms after onset of the cue. The cue and target remained on the screen until a response was provided or 1 s elapsed from target onset. Participants were required to indicate the position of the target as quickly and accurately as possible by pressing the “F” or “J” key. However, participants were told not to respond when the cue was a gray square (catch trial). The cue was considered congruent when the fish picture was directed toward the target position. All 103 pictures were presented twice, once each for the congruent and incongruent conditions. Thus, the cue congruency rate was 0.5, and the cue could not predict target position. There were also 24 catch trials, resulting in 230 trials in total.

### Results and Discussion

**FIGURE 5:**
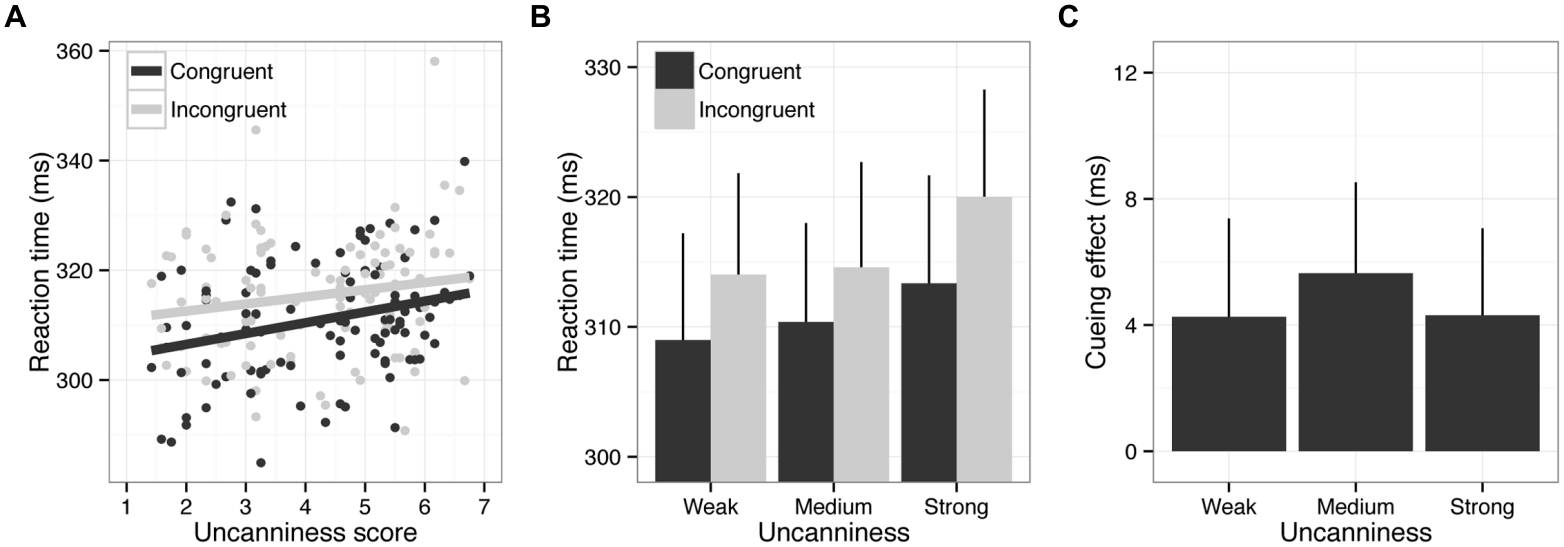
**Results of Experiment 2.** Error bars indicate SEM. **(A)** Scatter plot of RTs and uncanny scores. The lines depict the results of linear regression analysis. **(B)** RTs as a function of strength of uncanniness and cue congruency. Error bars indicate SE of mean (*N* = 25). **(C)** Cueing effects (difference in RTs between the congruent and incongruent conditions) as a function of strength of uncanniness. Error bars indicate SE of mean (*N* = 25).

Incorrect responses (2.02%) and outliers (0.80%) were excluded from the RT analysis. **Figure 5** depicts the results of Experiment 2. The results of the two-way repeated measures ANOVA showed significant main effects of uncanniness, *F*(2,48) = 5.11, *p* < 0.05, $\eta_{p}^{2}$ = 0.18, and cue-congruency, *F*(1,24) = 5.71, *p* < 0.05, $\eta_{p}^{2}$ = 0.19. However, no interaction between these factors was observed, *F*(2,48) = 0.25, *p* = 0.78, $\eta_{p}^{2}$ = 0.01. Paired comparisons showed that RTs in the strong uncanny condition were significantly longer relative to those of other conditions. These results were not attributable to the speed-accuracy tradeoff, as uncanniness did not affect the error rate, *F*(2,48) = 0.17, *p* = 0.77, $\eta_{p}^{2}$ = 0.01.

These results indicated that the presentation of uncanny stimuli led to longer RTs, even when the stimulus was task-irrelevant. The cueing effect was observed irrespective of the strength of the uncanniness of the cue stimulus. These results are consistent with a previous study using standard faces and faces with the eyes blacked out (Chaminade and Okka, 2013). This implies that feelings of uncanniness did not modulate the cueing effect (e.g., attention orienting to the congruent location) but delayed the processing of speeded response tasks both explicitly (Experiment 1) and implicitly (Experiment 2).

## Experiment 3

We found that uncanniness delayed directional discrimination and target detection in the cueing task. Threatening stimuli capture visual spatial attention and facilitate the perceptual process (Fox et al., 2000; Ohman et al., 2001). As uncanniness was associated with negative emotion (Experiment 1), strong uncanniness could have led to feelings of threat. If this were the case, one possible explanation for the effects of uncanniness in Experiments 1 and 2 was that threating objects captured visual spatial attention and simultaneously interfered with other perceptual processes: directional discrimination of the stimulus and attentional reorienting to the peripheral target. In Experiment 3A, we investigated the correlation between the uncanniness and threat. In Experiment 3B, using a dot-probe task, in which participants indicated the location of a target as quickly as possible while uncanny and non-uncanny probes were presented immediately before the target, we examined whether uncanny stimuli would capture visual spatial attention.

### Methods

#### Experiment 3A

Seventeen volunteers (eight male and nine female, mean age was 21.8) were newly recruited for Experiment 3A. The experiment consisted of two sessions; uncanniness rating and threat rating. The participants were asked to rate the uncanniness or the threat of a fish picture in each session. The session order was counterbalanced across participants. For each trial, a picture of a fish (640 × 640 pixel, 23° × 23°) was presented at the center of the screen. Scoring scales were presented below the stimulus picture. The participants viewed the pictures of fish freely and indicated their ratings using a mouse. After rating the fish according to the three questions, the subsequent fish picture appeared. We presented each participant with 180 pictures.

#### Experiment 3B

Twenty-four volunteers (13 male and 11 female, mean age was 21.6) were newly recruited for Experiment 3B. We used the ten most and ten least uncanny fish (hereinafter referred to as “uncanny fish” and “non-uncanny fish,” respectively). **Figure 3** shows the trial sequence. Each trial began with the presentation of a red fixation point at the center of a black screen for 500–1000 ms. Thereafter, two pictures (one randomly sampled from the uncanny fish and the other from the non-uncanny fish) were displayed above and below the fixation point for 300 ms (the size of each picture was 8.6° × 8.6°, and vertical eccentricity was 7.5°). The directions of the two fish pictures were the same and determined randomly for each trial. After a 100 ms blank display, a target dot (radius: 0.38°) appeared at the location of one of the two pictures and remained on the screen until a response was provided. Participants were required to indicate where the target dot appeared by pressing an arrow key as quickly and accurately as possible. In a catch trial, an instruction appeared in place of the target dot, and participants were asked to indicate the direction toward which the fish was oriented; therefore, participants were required to attend to the fish pictures. The subsequent trial began immediately. The target dot appeared equally often either at the location of the uncanny fish or of the non-uncanny fish. In total there were 120 target dot trials and 12 catch trials, all presented in random order.

### Results and Discussion

#### Experiment 3A

We analyzed the rating scores of 103 pictures that were used in the behavioral experiment. The mean uncanniness was 3.37 (SD = 1.15), while the mean threat was 3.80 (SD = 0.95). The correlation between uncanny and threat ratings was strong (*r* = 0.95, *p* < 0.001). However, the variance in the uncanny ratings for the fish pictures was larger than that of the threat ratings [paired Pitman–Morgan test: *t*(101) = 5.79, *p* < 0.001]. This indicated that, first, the features determining uncanniness and threat overlapped to a large extent, and second, the fish pictures varied more in terms of uncanniness than in terms of threat.

#### Experiment 3B

The correct rate in the catch trial was 98.6%, which suggested that participants attended to the fish pictures. Correct response rates for target detection did not differ between the uncanny and non-uncanny conditions [uncanny: 98.6% vs. non-uncanny: 98.7%, *t*(23) = 0.14, *p* = 0.89, *d* = 0.03]. Incorrect trials were removed from the RT analysis. RTs were also comparable between the uncanny and non-uncanny conditions [uncanny: mean = 362.9 ms, SE = 10.1 ms vs. non-uncanny: 362.1 ms, SE = 9.1 ms; *t*(18) = 0.23, *p* = 0.82, *d* = 0.05]. These results suggested that the uncanniness of fish stimuli did not modulate visual spatial attention. Therefore, it would be unlikely that the threat rather than uncanniness caused the delayed reaction in the previous experiments.

## General Discussion

The present study investigated the relationship between the uncanniness of visual stimuli and behavioral performance in speeded response tasks. Experiment 1 demonstrated that direction discrimination was slower for uncanny stimuli. Experiment 2 showed that the detection of a peripheral target was also slower when uncanny stimuli were presented in advance of the target as task-irrelevant cues. Experiment 3 revealed that uncanny stimuli did not capture visual spatial attention.

The main purpose of the present study was to investigate behavioral performance with uncanny visual stimuli. More specifically, we examined whether the visual presentation of uncanny stimuli would speed up or delay subsequent or concurrent perceptual processes. Essentially, our results suggested that a stronger feeling of uncanniness was accompanied by an increase in RTs. We found that uncanny fish pictures delayed speeded responses in both direction discrimination (Experiment 1) and spatial cueing tasks (Experiments 2). Note that body shape may covary with the uncanniness of the fish. If the uncanny fish had been more ambiguous in terms of direction, we should have observed a smaller cueing effect in the spatial cueing task. However, Experiment 2 showed that the cueing effects did not depend on the uncanniness of the fish. Therefore, difference in ambiguity, with respect to direction, could not account for the increase in RTs in the directional discrimination in Experiment 1. The results of the cueing experiment (Experiment 2) were partly consistent with a previous study (Chaminade and Okka, 2013). They presented human faces and humanoid faces as cue stimuli and found that responses were slower when the faces’ eyes were blacked out. One of the explanations they proposed was that the blacked-out eyes evoked feelings of uncanniness, which led to slower responses due to prediction error (Saygin et al., 2012). While Chaminade and Okka (2013) only investigated the cueing task, we showed that direct response toward uncanny stimuli was also delayed (Experiment 1). Therefore, the increase in RTs with uncanny stimuli was a general effect.

The underlying neural mechanisms remain unclear. One possible explanation could be threat-induced attentional capture. As feelings of uncanniness are often associated with fear (MacDorman and Ishiguro, 2006), uncanny stimuli could have captured visual spatial attention in a similar manner to that of threatening stimuli (Fox et al., 2000; Ohman et al., 2001). However, the results of Experiment 3 indicated that, unlike threatening stimuli, uncanny stimuli did not capture spatial attention. Experiment 3A implied that the difference in threat between the non-uncanny and uncanny fish would have been too small to influence threat-related attentional capture. The results indicated that the effects of uncanniness observed in the present study reflected those of uncanniness *per se*, or more conservatively, the effects did not arise in response to threat.

If threat-induced attentional capture is of no consequence, how did uncanny stimuli delay perceptual processes? Although uncanniness and delay in perceptual process were correlated, the causal relationship was unclear. Saygin et al. (2012) proposed that prediction error, resulting from a mismatch between appearance and motion, was the cause of the uncanny valley phenomenon. Yamada et al. (2012) found that morphed pictures (e.g., morphing between real and stuffed human beings) were disliked and difficult to categorize. They therefore proposed that the uncanny valley phenomenon was associated with categorical ambiguity; however, categorical ambiguity may also be a variant of prediction error, as we rarely encounter such morphed pictures showing categorical ambiguity. Therefore, their appearance is contrary to our general prediction regarding what faces will look like. Differences between prediction and input (i.e., prediction error) lead to greater neural activity (e.g., Summerfield et al., 2008; Larsson and Smith, 2012; Saygin et al., 2012), which implies that prediction error would require more cognitive and perceptual resources and could delay concurrent or subsequent perceptual processes. At the same time, prediction error also evoked the activities in the brain region related to the emotional processes (e.g., amygdala, Cheetham et al., 2011). Taken together, our interpretation was that prediction error led to feelings of uncanniness in the domain of emotional effects and simultaneously to delayed response in the domain of behavioral effects. This scheme may be related to the more general framework of attention and arousal for negative stimuli (Yechiam and Hochman, 2013). Presentation of negative stimuli sometimes leads to increases in RTs (Derryberry, 1991; Leppänen et al., 2003). Yechiam and Hochman (2013) attributed this effect to a more elaborate process, in which negative stimuli accompany an increase in general attention and arousal. This would fit the prediction error model, as prediction error would be submitted to the more elaborate process to modify the internal prediction template.

Another possibility could be based on familiarity. In studies exploring the uncanny valley, uncanniness is often associated with unfamiliarity (MacDorman and Ishiguro, 2006; Yamada et al., 2012). Similarly, uncanniness and familiarity ratings were strongly associated in the pilot experiment. In the visual search tasks, detecting an unfamiliar target among familiar distractors is more efficient than detecting a familiar target among unfamiliar distractors. Some studies proposed a theory in which processing familiar distractors is easier than is processing unfamiliar distractors (Wang et al., 1994; Shen and Reingold, 2001; Wolfe, 2001; Saiki et al., 2005). According to this theory, delayed response to uncanny stimuli may have occurred due to the unfamiliarity of uncanny stimuli.

The uncanny fish were considered atypical and rated as unfamiliar. While observers predicted the appearance of fish, uncanny fish did not match their predictions. This is a similar situation to that of the uncanny valley phenomenon, wherein a humanoid moves in a manner contrary to observers’ predictions. However, we should consider whether feelings of uncanniness regarding the fish and those involving humanoid robots in the uncanny valley are the same. Although we believe that these two feelings almost overlap, further studies investigating feelings of uncanniness and their relationship with behavioral performance, using a wide range of stimulus categories, are necessary to address this issue.

## Conclusion

The present study demonstrated the relationship between behavioral performance and feelings of uncanniness. Visual stimuli that evoke strong feelings of uncanniness turn out to delay speeded response. Importantly, this modulation appeared to be independent from the feeling of threat. The present study only examined speeded manual responses, but relationships between uncanniness and other behavioral effects (e.g., memory, avoidance behavior, or decision making) remain unclear. Further research is required to provide a more complete picture as to behavioral performance and feelings of uncanniness.

## Conflict of Interest Statement

The authors declare that the research was conducted in the absence of any commercial or financial relationships that could be construed as a potential conflict of interest.

## Footnotes

1 We used Japanese word “Bukimi” for the question of eeriness (uncanniness).
